# Salvage use of tissue plasminogen activator (tPA) in the setting of acute respiratory distress syndrome (ARDS) due to COVID-19 in the USA: a Markov decision analysis

**DOI:** 10.1186/s13017-020-00305-4

**Published:** 2020-04-20

**Authors:** Rashikh Choudhury, Christopher D. Barrett, Hunter B. Moore, Ernest E. Moore, Robert C. McIntyre, Peter K. Moore, Daniel S. Talmor, Trevor L. Nydam, Michael B. Yaffe

**Affiliations:** 1 Division of Transplant Surgery, Department of Surgery, University of Colorado Denver, Denver, CO USA; 2 Koch Institute for Integrative Cancer Research, Center for Precision Cancer Medicine, Departments of Biological Engineering and Biology, Massachusetts Institute of Technology, Cambridge, MA USA; 3 Division of Acute Care Surgery, Trauma and Surgical Critical Care, Department of Surgery, Beth Israel Deaconess Medical Center, Harvard Medical School, Boston, MA USA; 4 Ernest E Moore Shock Trauma Center at Denver Health, Department of Surgery, Denver, CO USA; 5 Department of Medicine, University of Colorado Denver, Denver, CO USA; 6 Department of Anesthesia, Critical Care and Pain Medicine, Beth Israel Deaconess Medical Center, Harvard Medical School, Boston, MA USA

**Keywords:** COVID-19, Acute respiratory distress syndrome (ARDS), Tissue plasminogen activator (tPA), Pulmonary failure, Fibrinolysis, Markov

## Abstract

**Background:**

COVID-19 threatens to quickly overwhelm our existing critical care infrastructure in the USA. Systemic tissue plasminogen activator (tPA) has been previously demonstrated to improve PaO_2_/FiO_2_ (mmHg) when given to critically ill patients with acute respiratory distress syndrome (ARDS). It is unclear to what extent tPA may impact population-based survival during the current US COVID-19 pandemic.

**Methods:**

A decision analytic Markov state transition model was created to simulate the life critically ill COVID-19 patients as they transitioned to either recovery or death. Two patient groups were simulated (50,000 patients in each group); (1) Patients received tPA immediately upon diagnosis of ARDS and (2) patients received standard therapy for ARDS. Base case critically ill COVID-19 patients were defined as having a refractory PaO_2_/FiO_2_ of < 60 mmHg (salvage use criteria). Transition from severe to moderate to mild ARDS, recovery, and death were estimated. Markov model parameters were extracted from existing ARDS/COVID-19 literature.

**Results:**

The use of tPA was associated with reduced mortality (47.6% [tTPA] vs. 71.0% [no tPA]) for base case patients. When extrapolated to the projected COVID-19 eligible for salvage use tPA in the USA, peak mortality (deaths/100,000 patients) was reduced for both optimal social distancing (70.5 [tPA] vs. 75.0 [no tPA]) and no social distancing (158.7 [tPA] vs. 168.8 [no tPA]) scenarios.

**Conclusions:**

Salvage use of tPA may improve recovery of ARDS patients, thereby reducing COVID-19-related mortality and ensuring sufficient resources to manage this pandemic.

## Introduction

The COVID-19 pandemic has quickly spread throughout the world and threatens to overwhelm our critical care bed supply in the USA in the upcoming weeks [1–3]. As of March 25nd, 2020, there were 435,000 cases of confirmed cases of COVID-19, expanding at an exponential rate with 40,000 patients in the past 24 h, and 20,000 deaths [4]. Estimates for the final death toll of COVID-19 if appropriate social distancing measures fail range from 200,000 to over 1,500,000 just in the USA [3]. Such a dire projection requires urgent, thoughtful, and creative action amongst clinicians.

Salvage use of therapeutics targeted at attenuating acute respiratory distress syndrome (ARDS) as a sequalae COVID-19-related mortality is of high interest [5, 6], and promising therapeutics may by invaluable for mitigating a large burden of advanced disease. A phase I clinical trial for salvage use in ARDS in 2001 reported significant improvements in PaO_2_/FiO_2_ when systemic tissue plasminogen activator (tPA) was given for patients with severe ARDS [7]. The mechanism of this finding is theorized to involve the dissolution of the extensive fibrin burden in the microcirculation and airway spaces in ARDS. There is growing evidence that lethal COVID-19 ARDS is associated with disseminated intravascular fibrin deposition [8, 9]. Consequently, there is revived interest in exploring the role of tPA in reducing ARDS-related mortality for COVID-19 patients who are eligible for salvage use therapeutics. A multicenter group from the USA has proposed that tPA could be an invaluable tool to reduce severe ARDS related to COVID-19 and act as a salvage technique to rescue patients when mechanical ventilation was not available [10]. This investigator cohort has set forward a treatment plan for the impending crisis.

However, it remains unclear to what extent the potential PaO_2_/FiO_2_ improvement of tPA would reduce ICU bed requirements and population-based mortality for the COVID-19 crisis. A long-term randomized clinical trial is not practical in the midst of the current pandemic. However, in our unprecedented setting, a decision analytic Markov model may offer guidance. Markov models use existing published data to project the movement of patients through simulated health states over time [11]. Markov models have previously been used by our group and have been increasingly applied to model healthcare challenges for which trial data may be lacking for the foreseeable future [12–15]. As such, a Markov model was created to estimate the impact of salvage use systemic tPA on ARDS/COVID-19 patients in addition to a more aggressive use for interventions at an earlier state of disease progression.

## Methods

A decision analytic Markov state transition model was created to simulate the life critically ill COVID-19 as they transitioned to either recovery or death. Two patient groups were simulated as follows: (1) patients received tPA immediately upon diagnosis of ARDS or (2) patients received standard therapy for ARDS. Medical decision-making software was utilized for the creation and computation of the model (DATA 3.5, TreeAge Software Inc, Williamstown, MA). Base case patients were defined as having a PaO_2_/FiO_2_ (P/F) of < 60 mmHg, as this approximates the initial (within 7 days) PaO_2_/FiO_2_ of current ARDS/COVID-19 patients who would be eligible for salvage use of therapeutics.

### Decision model structure (Fig. 1)

One hundred thousand patients were modeled in the two arms of the decision model (50,000 patients in each arm). The model began following the admission of a simulated ARDS/COVID-19 patient to the ICU. Patients in the tPA arm received therapy within 7 days of admission, while patients in the no tPA received standard ARDS treatment. Patients were followed as they transitioned through severe/moderate/mild ARDS, recovery, and death. Seven-day cycle lengths were used until all patients died or recovered.

**Fig. 1:**
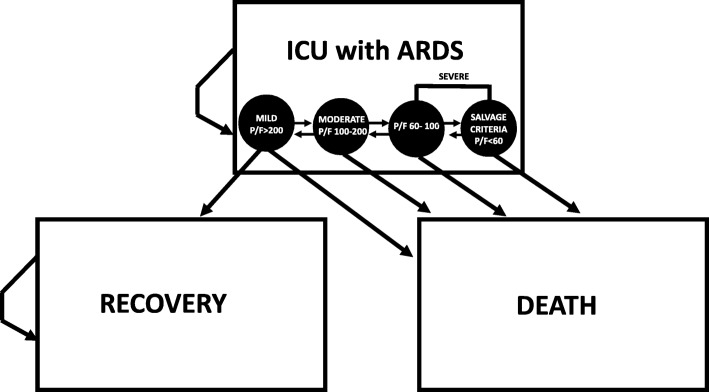
Decision model structure

### State transition probabilities (Table 1)

Transition probabilities were extracted from existing, published literature using MEDLINE electronic search. As has been previously described by Naughler et al., transition probabilities for various time periods were converted into rates per cycle with the actuarial method for 7-day cycle probabilities, as such [16]

$$ \mathrm{P}=1-{\exp}^{-\mathrm{rt}} $$

$$ \mathrm{Standard}\ \mathrm{rate}=\ln \left(1-P\right)/t $$

$$ \mathrm{Seven}\ \mathrm{day}\ \mathrm{transition}\ \mathrm{probability}=1-{\mathit{\exp}}^{\left[7\mathrm{xln}\left(1-\mathrm{P}\right)/\mathrm{t}\right]} $$

**Table 1:** State Transitions - Variable Table

Variable	Value	Reference
Severe to Moderate ARDS	45.0% in 7 days	Bellani et al. 2016 [17]
Moderate to Severe ARDS	12.7% in 7 days	Bellani et al. 2016 [17]
Mild to Moderate ARDS	25.8% in 7 days	Bellani et al. 2016 [17]
Mild to Severe ARDS	4.5% in 7 days	Bellani et al. 2016 [17]
Mild ARDS to Floor/Recovery	60% in 28 days	Bellani et al. 2016 [17]
Severe ARDS Mortality	40.9% in 28 days	Bellani et al. 2016 [17]
Moderate ARDS Mortality	35.0% in 28 days	Bellani et al. 2016 [17]
Mild ARDS Mortality	29.6% in 28 days	Bellani et al. 2016 [17]
Mean Presenting PaO_2_/FiO_2_ Ratio (mm Hg), ICU-COVID Patients	136 (IQR: 103-234)	Wang et al. 2020 [6]
PaO_2_/FiO_2_ Ratio (mm Hg), for Salvage Use tPA	60	
Mean Improvement PaO_2_/FiO_2_ Ratio (mm Hg), ICU-ARDS Patients	170 (IQR: 135-225)	Hardaway et al. 2001 [7]
Death or non-fatal disabling stroke following systemic tPA	0.6%	GUSTO-I Trial [18]

(*P* = probability of event; *exp* = base of natural logarithm; *r* = rate of event occurring; *t* = time period in days; *ln* = natural logarithm).

### Systemic tPA PaO_2_/FiO_2_ improvement

Natural history of ARDS was extracted from Bellani et al. [17]. To estimate improvement of PaO_2_/FiO_2_ following tPA, results from Hardaway et al. were applied [7]. It was assumed that patient’s response to tPA followed a normal distribution in regard to probability of transitioning from severe to moderate to mild ARDS. Standard scores were calculated using:

$$ z=\left(\mathrm{X}-\upmu \right)/\upsigma $$

(*z =* standard score; *X =* PaO_2_/FiO_2_ cut-off value (100 mmHg and 200 mmHg); *μ =* mean PaO_2_/FiO_2_ following treatment; *σ =* standard deviation).

Standard scores were converted to percentiles using table for one-sided standard normal distribution. The calculated transition probabilities were assumed to be independent of the initial ARDS class.

Mortality rate following systemic tPA was extrapolated from the GUSTO-I trial, whereby tPA use was associated with a 0.6% death or non-fatal debilitating stroke rate [18].

Our group has previously used the presented approach to transition probability creation in Markov decision analysis to estimate the impact of weight loss interventions for renal and liver transplant candidates [12–15].

### COVID-19 Imperial College Response Team projection

The Imperial College has recently published their projection of COVID-19 critical care bed utilization in various scenarios, no social distancing and optimal social distancing [19]. The impact of tPA on mortality was extrapolated (standard method) upon these projections to create population-based mortality estimates. Two scenarios were modeled as follows: (1) tPA given to only patients eligible for salvage use therapeutics and (2) all patients who presented to ICU with ARDS. Estimates for model 1 were extracted from the Wuhan COVID-19 experience [6].

## Results

### State transitions

**Fig. 2:**
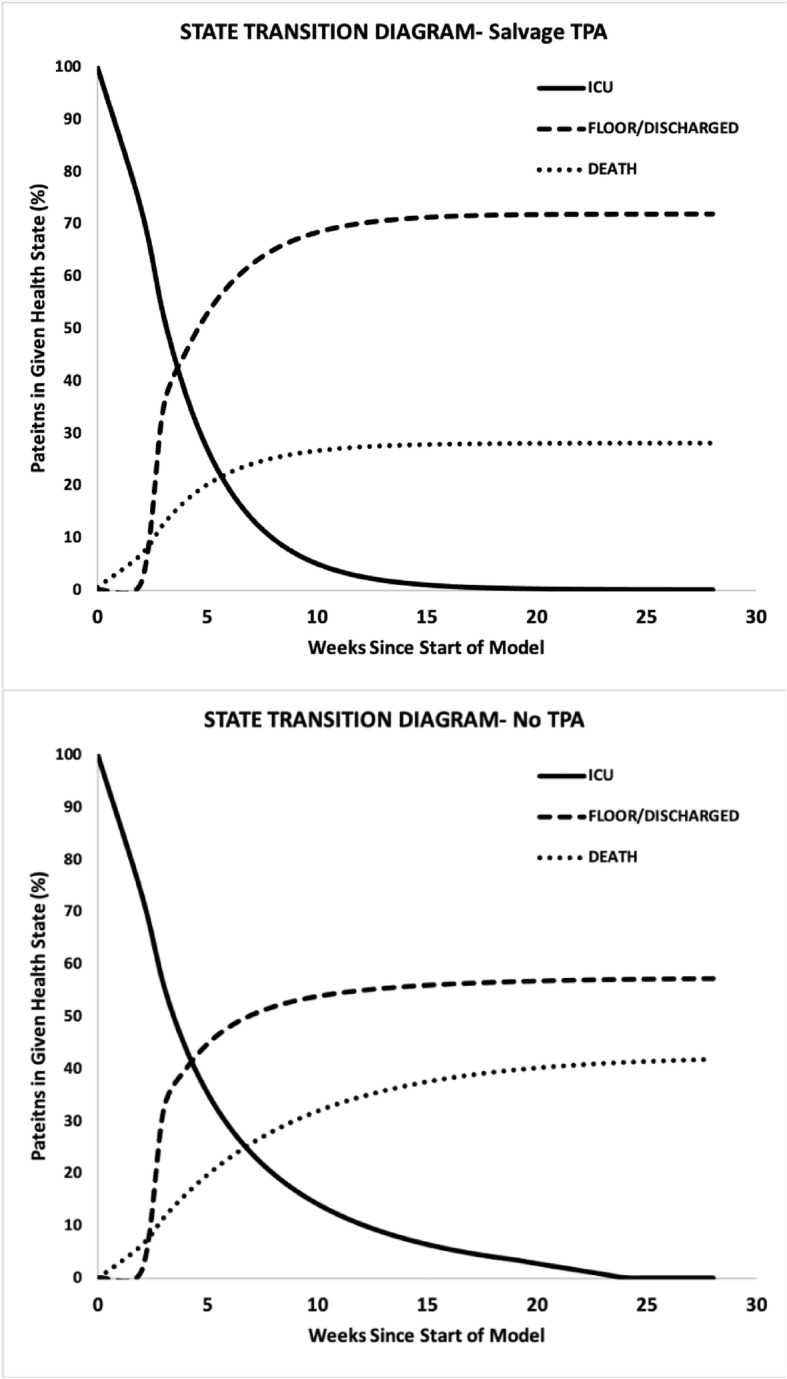
State transition diagram, salvage use tPA, and no tPA for patients with PF< 60 (**a**, **b**)

Patients were followed as they cycled through the model (Fig. 2a, b). For base case patients, all patients began in the severe (salvage use) ARDS health state, and transitioned through ARDS health states (mild, moderate, and severe), and then to either recovery or death. Compared to patients who did not undergo tPA treatment, patient who received salvage use tPA therapy transitioned to recovery (floor status/discharge) more quickly and more frequently. Correspondingly, the use of tPA was associated with reduced mortality (47.6% [tTPA] vs. 71.0% [no tPA]) at the conclusion of the model.

**Fig. 3:**
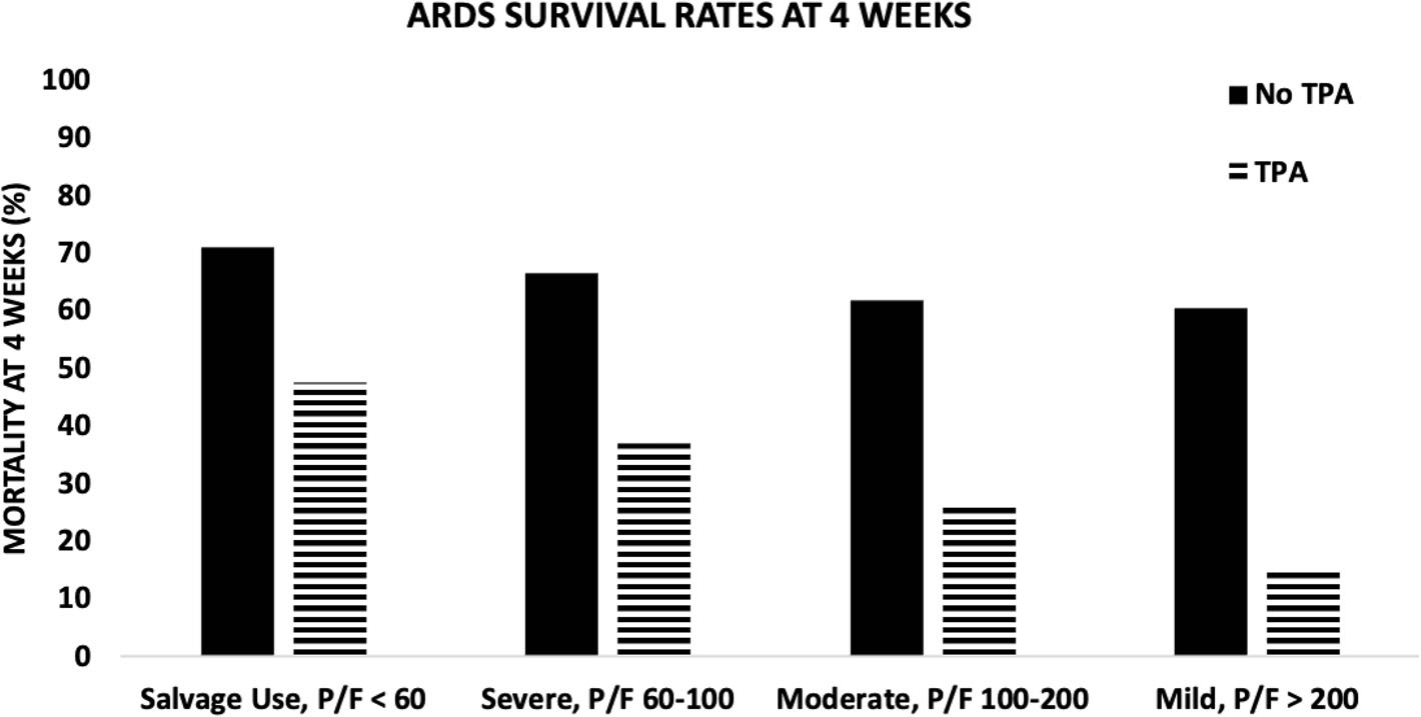
Survival by ARDS severity

A sensitivity analysis of initial (within 7 days) ARDS status (mild vs. moderate vs. salvage use criteria ARDS) was performed (Fig. 3). Utilization of tPA was estimated to significantly reduce 4-week mortality for initial mild (15.2% [tTPA] vs. 60.5% [no tPA]), moderate (26.4% [tTPA] vs. 62.0% [no tPA]), and salvage use criteria (47.6% [tTPA] vs. 71.4% [no tPA]) ARDS.

### Population-based mortality

**Fig. 4:**
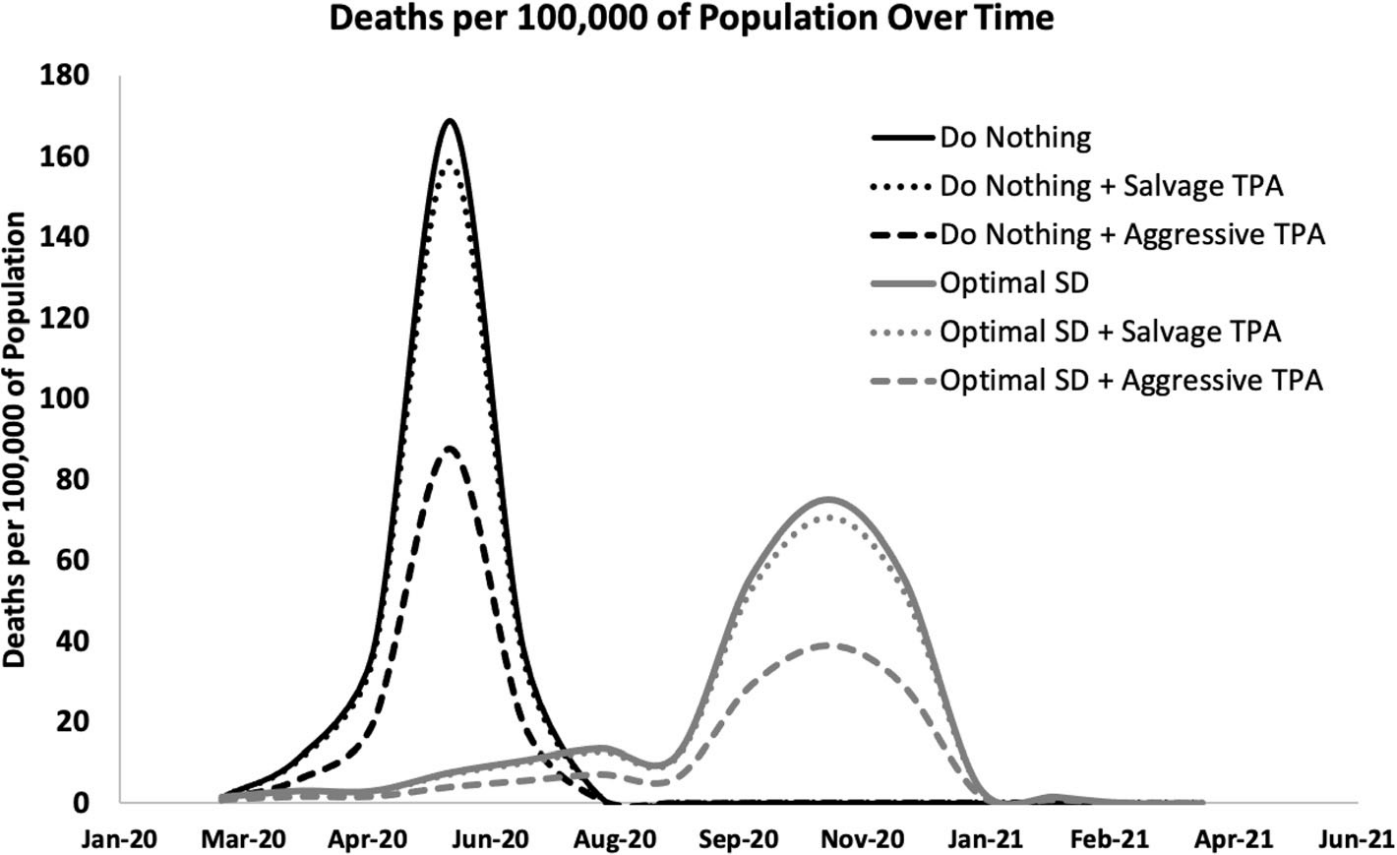
Population-based mortality over time

Results of base case were extrapolated to Imperial College COVID-19 Response Team projections of COVID-19 temporal disease burdens. When only utilized in a salvage use scenario, peak mortality (deaths/100,000 patients) was reduced for optimal social distancing (70.5 [tPA] vs. 75.0 [no tPA]) and no social distancing (158.7 [tPA] vs. 168.8 [no tPA]) scenarios. However, when expanded to the entire COVID-19 mechanically ventilated population, mortality was drastically reduced, again for both optimal social distancing (39.1 [tPA] vs. 75.0 [no tPA]) and no social distancing (87.7 [tPA] vs. 168.8 [no tPA]) scenarios (Fig. 4).

## Discussion

Current interest in utilizing tPA for ARDS patients represents a novel potential therapy to reduce mortality in the current COVID-19 pandemic. In our Markov model, salvage use was estimated to have minimal impact on survival. However, the more aggressive use of tPA with all stages of ARDS was estimated to significantly improve ventilatory requirements for such patients, thereby improving survival. Furthermore, patients required critical care beds for reduced amounts of time compared to current estimates with utilization of standard methods of ARDS treatment. This model offers hope and support, globally, for the growing number of centers considering salvage use of systemic tPA.

As Markov models rely on extrapolation, several weaknesses of the model should be addressed. Although Hardaway et al. demonstrated improvement in the P/F ratio with tPA administration in patients with severe ARDS P/F < 60%, it is not clear whether this effect would be sustained in COVID-19 patients, as tPA would theoretically reduce the sequalae of ARDS, but not lead to improved viral clearance. As such, long-term efficacy is not proven at the current time. Additionally, the model is based on data from the Wuhan experience, a population whose demographics approach but are not identical to those in the USA. Furthermore, in the no TPA arm, transition probabilities for ARDS progress/improvement and associated mortality were assumed to mirror non-COVID-19 diseases—an assumption for which insufficient data exists to validate.

## Conclusions

Despite the limitations of this Markov model, the reality of the impeding global disaster with the COVID-19 is coming true. Prior models of the overwhelming exponential growth of disease in Italy with saturation of the medical system have almost nightmarishly exceeded expectations [20]. Italy now has a mortality rate of > 9% with more overall deaths than China [4]. Protocols for salvage use of systemic tPA are in planning at several centers in the USA [10]. If salvage use has efficacy in improving pulmonary function in ARDS related to COVID-19, a quick response to implement a clinical trial would be necessary to generate sufficient evidence for a validated treatment to effectively reduce ARDS-related mortality. At the rate of growth of this pandemic, time is the enemy. Without effective social implementation to curtail the rapid spread of this virus, tPA may never have the opportunity to be used beyond salvage use.

## Data Availability

Not applicable all data currently online

## Abbreviations

tPA: Tissue plasminogen activator
ARDS: Acute respiratory distress syndrome
P/F: Ratio of partial pressure of blood oxygen level to percent of oxygen inhaled
